# Functional decline, long term symptoms and course of frailty at 3-months follow-up in COVID-19 older survivors, a prospective observational cohort study

**DOI:** 10.1186/s12877-022-03197-y

**Published:** 2022-06-30

**Authors:** Simon Prampart, Sylvain Le Gentil, Marie Laure Bureau, Claire Macchi, Caroline Leroux, Guillaume Chapelet, Laure de Decker, Agnes Rouaud, Anne Sophie Boureau

**Affiliations:** 1 Department of Geriatrics, Nantes University Hospital, Boulevard Jacques Monod, 44093 Nantes, France; 2 Université de Nantes, CHU Nantes, CNRS, INSERM, l’institut du thorax, F-44000 Nantes, France

**Keywords:** COVID-19, Functional decline, Activity of daily living, Long term symptoms, Frailty, Older patients, Geriatric care

## Abstract

**Background:**

Aging is one of the most important prognostic factors increasing the risk of clinical severity and mortality of COVID-19 infection. However, among patients over 75 years, little is known about post-acute functional decline.

**Objective:**

The aim of this study was to identify factors associated with functional decline 3 months after COVID-19 onset, to identify long term COVID-19 symptoms and transitions between frailty statesafter COVID-19 onset in older hospitalized patients.

**Methods:**

This prospective observational study included COVID-19 patients consecutively hospitalized from March to December 2020 in Acute Geriatric Ward in Nantes University Hospital. Functional decline, frailty status and long term symptoms were assessed at 3 month follow up. Functional status was assessed using the Activities of Daily Living simplified scale (ADL). Frailty status was evaluated using Clinical Frailty Scale (CFS). We performed multivariable analyses to identify factors associated with functional decline.

**Results:**

Among the 318 patients hospitalized for COVID-19 infection, 198 were alive 3 months after discharge. At 3 months, functional decline occurred in 69 (36%) patients. In multivariable analysis, a significant association was found between functional decline and stroke (OR = 4,57, *p* = 0,003), history of depressive disorder (OR = 3,05, *p* = 0,016), complications (OR = 2,24, *p* = 0,039), length of stay (OR = 1,05, *p* = 0,025) and age (OR = 1,08, *p* = 0,028). At 3 months, 75 patients described long-term symptoms (49.0%). Of those with frailty (CFS scores ≥5) at 3-months follow-up, 30% were not frail at baseline. Increasing frailty defined by a worse CFS state between baseline and 3 months occurred in 41 patients (26.8%).

**Conclusions:**

This study provides evidence that both the severity of the COVID-19 infection and preexisting medical conditions correlates with a functional decline at distance of the infection.

This encourages practitioners to establish discharge personalized care plan based on a multidimensional geriatric assessment and in parallel on clinical severity evaluation.

**Supplementary Information:**

The online version contains supplementary material available at 10.1186/s12877-022-03197-y.

## Key messages

- In older frail patients, functional decline after COVID-19 infection was associated with age, preexisting medical conditions (history of stroke, depressive semiology), COVID-19 complications and length of hospital stay.
- After 3-months follow-up, half of the patients had long term symptoms and 27% worsened frailty status.

## Background

The severe acute respiratory syndrome coronavirus 2 (SARS-CoV-2) is a new beta-coronavirus detected in China in the late 2019. The total number of worldwide COVID-19 confirmed cases is of 310 million leading to 5.5 million deaths [1]. Among prognostic factors increasing the risk of clinical severity and mortality, aging is one of the most important [2–4]. Indeed, hospital mortality ranges from less than 5% among patients younger than 40 years to more than 60% for patients aged of 80 or older [5]. Multimorbidity and frailty are frequent conditions in older patients and worsen COVID-19 prognosis [6–9].

Beside high risk of severe acute disease and mortality, post-acute functional status is also a major concern for older patients. Indeed, hospitalization for an acute disease or infection can lead to functional impairment. For example community acquired pneumonia or severe infection lead to an increased limitation in activities of daily living, and impact on the health-related quality-of-life in the post-discharge period [10–12]. After COVID-19, 27 to 54% of patients described functional decline [13, 14]. Specific factors associated with this functional decline in older adults are unknown. Indeed, post-acute functional decline could be secondary to pre-sepsis health status, clinical severity and to persistent symptoms [13, 15, 16]. Recent cohorts have described post-COVID syndrome, also known as Long-COVID, defined as the association of various physical and neuropsychiatric symptoms that persist for more than 12 weeks after COVID-19 onset without an alternative explanation [17]. In patients younger than 75 years old, up to 80% developed one or more long-term symptoms after COVID-19 onset [18]. Those impairments seem to be more important in patients with more severe disease [19–22]. In this context, we could therefore infer that severe COVID-19 disease may theoretically index worse functional decline in frail older patients. Furthermore, if older survivors experienced functional decline or developed long-COVID symptoms, they may also be at high risk for increasing frailty [23]. Indeed, frailty is common among survivors of critical illness, either newly acquired or worsened frailty [24]. However, the transition between frailty state after a COVID-19 is currently unknown.

Therefore, the aim of this prospective study was to identify in older hospitalized patients i) the association between COVID-19 severity and worse functional decline 3 months after COVID-19 onset, ii) incidence and patterns of long term COVID-19 symptoms, iii) transitions between frailty states after COVID-19 onset especially in patients with functional decline.

## Methods

### Study design and study population

From March 11th to December 31st 2020, we conducted a longitudinal observational cohort study including all patients aged 75 years and older, consecutively admitted in Acute Geriatric Wards of Nantes University Hospital, in France, for a confirmed SARS-Cov-2 infection. Patients hospitalized in that ward were patients aged 75 and older with ICU admission denied or not required following the French guidance published during that inclusion period [25].

The inclusion criteria were i) 75 years-old and over, ii) SARS-CoV-2 infection confirmed with Polymerase Chain Reaction (PCR) or a thoracic CT-scan, iii) 3 months’ follow-up as it is usually done.

Prior to any data collection, the investigator presented the interest of the study and ensured an oral consent. The study was approved by the local ethics committee, *Groupe Nantais d’Ethique dans le Domaine de la Santé* (GNEDS), which waived the need for written informed consent in keeping with legislation on analyses of anonymized data. All research was performed in accordance with the ethical standards set forth in Helsinki declaration (1983).

### Data collection

In consistency with standard care and management of acute geriatric ward, medical investigators collected the study data with the patient and/or the caregiver interview at the admission and using structured electronic case report. Collected data included sociological and demographic variables (age, sex, previous institutionalization), body mass index (BMI), comorbidities (Charlson Comorbidity Index), number of medications, the presence of cognitive disorder, previous fall in the precedent year, clinical and laboratory findings at hospital admission; duration of COVID-19 symptoms; radiological findings; specific treatments as glucocorticoid and presence and severity of organ dysfunction according to the Sequential Organ Failure Assessment (SOFA) score (collected data detailed in supplementary file).

The functional status and instrumental status respectively was assessed by the Katz Activities of Daily Living scale (ADL) including bathing, dressing, toileting, transferring, continence, feeding and the 4 items and by Instrumental Activities of Daily Living simplified scale (IADL) including ability to use the telephone, mode of transportation, responsibility for own medications, ability to handle finances. ADL ranges from 0 (severe functional impairment) to 6/6 (full functional status); and IADL from 0 (dependency) to 4/4 (full functional status) [26, 27] .The functional status scores were calculated with the patients’ abilities 15 days before admission, defined as the pre-admission ADL. Frailty was assessed using the Clinical Frailty Scale (CFS) by trained geriatricians [28]. Baseline frailty was defined according to pre-COVID health conditions, characterized per information regarding the 2 weeks before the infection. As previously published, CFS score–based frailty groups were: fit if the CFS score was1–3, vulnerable if the CFS score was 4, frail if the CFS score was 5–7 and severely frail if CFS was 8–9 [24, 29]. Other geriatrics parameters were collected such as: falls in the precedent year, Body Mass Index (BMI). Baseline demographics and comorbidities; clinical and laboratory findings at hospital admission; history of the symptoms; radiological findings; specific treatments as glucocorticoid were collected and are detailed in supplementary data.

### Follow up data

Three months after discharge, a face-to-face in-hospital visit (or by structured follow-up telephone interviews with participants or their proxy if they were unable to move around) was conventionally conducted by a geriatrician. The following outcome variables were collected: functional assessment (ADL, IADL), frailty (CFS), nutritional assessment (loss of weight), hospital readmission, persistent COVID-19 symptoms, fall, modified medical research council dyspnea scale (MMRC). Both face-to-face visit or phone interview were conducted with the patient, family careers or care home staff. These variables were assessed as described above.

### Study end points

The main outcome was the functional decline at 3 months follow-up defined as the loss of at least 1 point on ADL scale. The secondary outcomes were evaluation of long term COVID-19 symptoms and the transition between frailty state based on transition between CFS categories between admission and 3 months.

### Statistical analysis

Quantitative data are expressed as mean (SD) or median (25th – 75th percentile) according to their distribution, and were compared using Student’s T test or Mann Whitney U test, respectively. For categorical variables, data were presented as frequencies and percentages (%) and were compared using χ2 test or Fisher’s exact test depending on size (> 5) between the two groups (i.e. alive vs. dead). Multivariable logistic regression was used to identify factors associated with functional decline. The OR and the confidence intervals (CI) are presented in the charts. Variables in the models were checked for collinearity. All baseline variables with a *p*-value< 0.10 in univariable analysis were included in multivariable logistic regression. To previous studies and to permit an analysis taking care of the largest number of potential confounders, we incorporated in our model series of variables, including cognitive performance, functional status, comorbid conditions and SOFA scale. A *p*-value < 0.05 was considered as statistically significant, without correction for multiple testing. We did not input missing data in any of our analyses.

To illustrate transitions between frailty states from baseline to 3-months follow-up, we constructed alluvial flow diagrams.

All analyses were performed without imputation, using statistical software R, version 4.0.3 (R Foundation for Statistical Computing, Vienna, Austria).

The datasets generated and analyzed during the current study are not publicly available in accordance with data protection regulations but are available from the corresponding author on reasonable request.

### Sample size calculation

The sample size calculation was not possible at the time we started the study. Indeed, 3-months functional decline in COVID-19 patients was unknown when we started the study.

## Results

### Study population and baseline characteristics

**Fig. 1:**
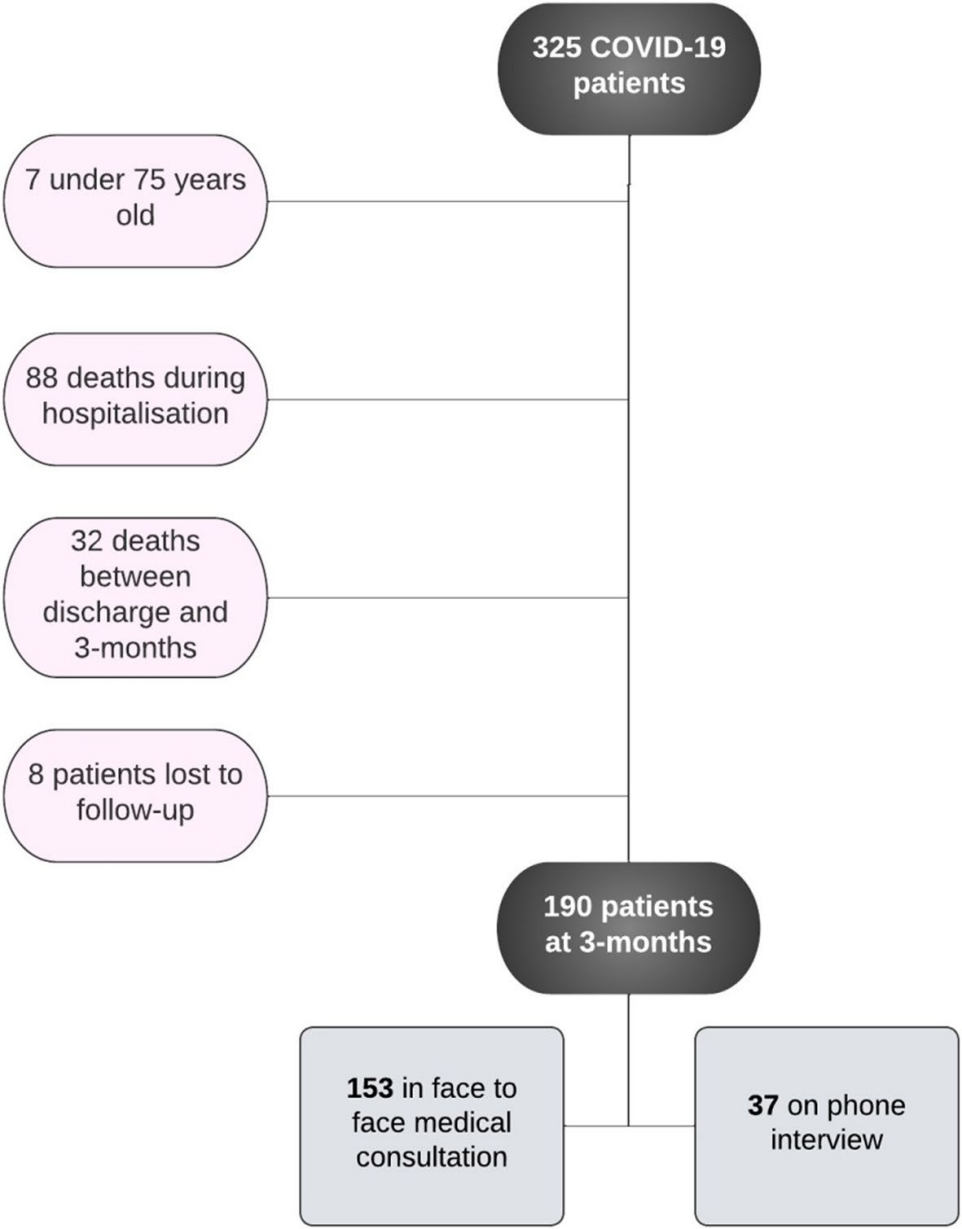
Flow chart of the study population

Between March the 11st 2020 and December the 31th 2020, 318patients aged 75 and older were hospitalized for COVID-19, in the Acute Geriatric Ward of Nantes University Hospital and were candidate for the study. Among them, 120 patients (37%) died in hospital or after hospital discharge. Therefore, 198 patients were alive at 3 months (62.3%) of whom 8 patients (4%) were lost to follow-up (Fig. 1).

In the study population corresponding to survivors, median age was 86 (IQR: 82–90) and 36% were male (Table 1). Median CCI was 2 (IQR: 1–3). Median pre admission ADL and IADL were respectively 5/6 (IQR: 3–6) and 1/4 (IQR: 0–3). Median pre admission CFS was 5 (IQR: 3–6). Median length of stay was 11 days (IQR: 7–17). Median maximal oxygen supply was 2 L/min (IQR: 0–4) (FiO2 = 0.26). One hundred and thirty-three patients (67%) had at least one complication: eighty-five (42%) had a secondary infection, 12 (6%) had a thrombosis and 29 (14.6%) had an acute kidney failure. Eighty patients (40%) received corticosteroid drugs, 116 (59%) needed supplemental oxygen therapy. Two patients were transferred in ICU. Eighty-six patients (56%) were discharged home, 72 patients (43%) to rehabilitation center. Twenty-nine patients (23%) were hospitalized again before 3-months follow-up. Characteristic of survivors and dead patients are detailed in Table 1.

**Table 1:** Baseline study population characteristics

	COVID-19 Survivors ***N*** = 190	No ADL decline ***N*** = 121	ADL decline ***N*** = 69	***P*** value
**Demographic and anthropometric**				
Male	68 (35.8%)	46 (38.0%)	22 (31.8%)	0.400
Age (years)	86 (82–90)	85 (82–89)	88 (84–92)	0.003
Nursing home	72 (38.0%)	43 (35.5%)	29 (42.0%)	0.410
BMI (kg/m^2^)	24.6 (21.9–28.2)	26.1 (25.2–28.9)	23.7 (21.1–26.7)	0.020
**Comorbidities**				
CCI	2 (1–3)	2 (1–3)	3 (1–4)	0.100
Chronic respiratory insufficiency	11 (5.8%)	7 (5.8%)	3 (4.3%)	0.700
Stroke	35 (18.4%)	12 (9.9%)	23 (33.3%)	< 0.001
Diabetes	32 (16.8%)	18 (14.8%)	14 (20.3%)	0.330
Coronaropathy	50 (26.3%)	32 (26.4%)	18 (26.1%)	0.990
Hypertension	145 (76.3%)	83 (68.6%)	56 (81.1%)	0.060
Chronic kidney disease stage 4 or 5	18 (9.5%)	13 (10.7%)	5 (7.2%)	0.420
**Geriatric assessment**				
Pre admission ADL	5 (3.0–6.0)	5 (3.0–6.0)	4.5 (3.5–5.5)	0.160
Pre admission IADL	1 (0–3.0)	1 (0–4.0)	1 (0–1.0)	< 0.001
Pre admission CFS	5 (3–6)	4 (3–6)	5 (4–6)	0.030
Neurocognitive disorder	95 (50%)	52 (42.9%)	43 (62.3%)	0.010
Prior fall	94 (49.7%)	52 (42.9%)	42 (60.9%)	0.007
Number of medications	7 (5.0–10.0)	7 (5.0–9.0)	8 (6.0–12.0)	0.007
Depressive semiology	36 (18.9%)	18 (14.8%)	18 (26.1%)	0.060
**Clinical features**				
Respiratory signs	146 (76.8%)	90 (74.4%)	56 (81.1%)	0.320
Fever	102 (53.7%)	61 (50.4%)	41 (59.4%)	0.210
Delirium	48 (25.2%)	27 (22.3%)	21 (30.4%)	0.200
Dehydration	58 (30.5%)	30 (24.8%)	28 (40.6%)	0.020
Diarrhea	46 (24.2%)	24 (19.8%)	22 (31.9%)	0.060
SOFA score (hospital admission)	0 (0–1)	0 (0–1)	1 (0–2)	0.370
Maximal oxygen supply (L/min)	2 (0–4)	2 (0–4)	3 (0–5)	0.260

### Functional decline

Functional status was assessed for 190 patients (153 in face to face medical consultation and 37 on phone interview). Three months’ functional decline occurred in 69 patients (36%). In this sub-group, the mean ADL decrease was of 1.5 points (IQR: 1–2.5) out of the 6 points of the ADL scale. Other 3 months’ outcomes are detailed in Table 2.

**Table 2:** Outcomes at 3 months after discharge

	Survivors ***N*** = 190	No ADL decline ***N*** = 121	ADL decline ***N*** = 69	Missing values	***P*** value
Length of hospital stay	10 (6–16)	9 (6–15)	14 (9–20)	0	< 0.001
Rehabilitation center	72 (42.8%)	32 (31%)	40 (61%)	22	< 0.001
Discharge hospital at home	86 (55.7%)	67 (68%)	19 (35%)	37	< 0.001
Readmission	29 (23%)	15 (19%)	24 (29%)	64	0.200
Long term COVID symptoms	75 (39%)	50 (41%)	25 (36%)	37	0.860
Falls	24 (24%)	14 (21%)	10 (29%)	–	0.400
MMRC	0 (0–1)	0 (0–1)	0 (0–3)	99	0.200
No transition in frailty status	104 (54.7%)	76 (62.8%)	28 (40.6%)	49	< 0.001
Worse frailty status	41 (21.6%)	12 (9.9%)	29 (42.1%)	49	< 0.001
Median CFS increase	1 (0–1)	0 (0–1)	1 (1–2)	41	< 0.001
Weight variation (%, mean ± SD)	−1.4 ± 5.4	−1.3 ± 4.3	−1.5 ± 6.1	71	0.600

*ADL* Activities of daily living, *CFS* Clinical Frailty Scale, *MMRC* Modified Medical Research Council Dyspnea Scale

In univariable logistic regression analysis, a significant association was found between functional decline and age (OR = 1,08 CI [1,02; 1,15], *p* = 0,005); history of stroke (OR = 4,54 CI [2,12; 10,2], *p* < 0,001); preadmission IADL (OR = 0,64 CI [0,50; 0,81], *p* < 0,001); preadmission CFS (OR = 1,23 CI [1,02; 1,50], *p* = 0,028); cognitive disorder (OR = 2,12 CI [1,15; 3,95], *p* = 0,015); prior fall (OR = 2,52 CI [1,29; 5,07], *p* = 0,006); number of medications (OR = 1,13 CI [1,03; 1,23], *p* = 0,006); BMI (OR = 0,92 CI [0,86; 0,99], *p* = 0,028); dehydration (OR = 2,07 CI [1,10; 3,92], *p* = 0,024); length of stay (OR = 1,06 CI [1,02; 1,09], *p* = 0,001); secondary bacterial infection (OR = 1,85 CI [1,01; 3,42], *p* = 0,047); occurrence of a complications (such as thrombosis OR/AND acute kidney failure with dehydration OR/AND diarrhea OR/AND secondary infection) (OR = 2,48 CI [1,24; 5.22], *p* = 0,012)(Table 3 and Table 1S in Supplemental Data).

**Table 3:** Multivariable analysis of factors associated with functional decline

	OR [95%CI]	***P*** value
Age	1.09 (1.01, 1.18)	0.031
Male	1.17 (0.44, 3.11)	0.751
Pre-admission CFS	1.06 (0.73, 1.36)	0.780
SOFA	0.95 (0.67, 1.31)	0.751
CCI	0.98 (0.78, 1.23)	0.884
Stroke	4.71 (1.47, 16.54)	0.010
Depressive semiology	3.53 (1.32, 9.89)	0.013
Hypertension	1.15 (0.43, 3.18)	0.583
Cognitive disorder	1.99 (0.77, 5.20)	0.155
Previous Falls	1.66 (0.67, 4.16)	0.275
Weight	0.99 (0.96, 1.01)	0.326
Complications^a^	2.02 (1.02, 4.86)	0.045
Length of stay	1.06 (1.01, 1.011)	0.015

*CFS* Clinical Frailty Scale, *CCI* Charlson Comorbidity Index, *SOFA* Sequential Organ Failure Assessment

^a^Complications: Thrombosis or acute kidney failure with dehydration or diarrhea or secondary infection

After analysis, pre-admission CFS, pre-admission ADL and pre-admission IADL were found to be correlated, thus we chose to keep the pre-admission CFS only for the multivariable analysis. Weight measure was included instead of the BMI in the multivariable analysis because of an important number of missing data for BMI assessment. Because of their effect on functional decline described in previous published studies, CCI, SOFA score and sex were included in the statistical model.

In multivariable analysis, a significant association was found between functional decline and stroke (OR = 4,57 CI [1,71; 13,03], *p* = 0,003), history of depressive disorder (OR = 3,05 CI [1,24; 7,75], *p* = 0,016), complications (OR = 2,24 CI [1,05; 4,92], *p* = 0,039), length of stay (OR = 1,05 CI [1,01; 1,09], *p* = 0,025) and age (OR = 1,08 CI [1,01; 1,16], *p* = 0,028) (Table 3).

### Long term symptoms and CFS evolution at 3 months’ follow-up

This analysis was done in the population with face-to-face medical interview: 153 patients out of the 198 survivors. At 3 months, 75 patients described long-term symptoms (49%) defined as a new or increased symptoms occurring since COVID-19 hospitalization. These symptoms were fatigue (47, 31%), breathless (24, 16%), loss taste (13, 9%), joint pain (10, 7%), muscle pain (8, 5%), diarrhea or nausea or abdominal pain (8, 5%), loss smell (8, 5%), headache (5, 3%), skin rash (3%). In patients with dyspnea, the median MMRC score was 2 (IQR: 1–3). Other symptoms more frequently described in older population were also described as: new memory difficulties or progression of neurocognitive disorders (60, 39%), walking disabilities or falls (25, 16%), depressive symptoms (14, 9%), loss of appetite or weight loss (11, 7%) (Table 2). The presence of long-term symptoms was not associated with functional decline in univariable analysis (OR = 0.84, CI [0.36–1.98], *p* = 0.86) (Table 1S in Supplemental Data).

In this sub-group, the prevalence of frailty increased following COVID-19 hospitalization. At pre-admission, there were76 of 153 patients (49.6%) with frailty (i.e., CFS score ≥ 5). At 3-months, 101 (66.0%) were frail. Of the 101 patients with frailty at 3-month follow-up, 30 (30%) were not frail (i.e., CFS score < 5) at pre-admission. Overall, the median increase in CFS scores was 1 (IQR: 0–2) between pre-admission and 3 months after discharge.

**Fig. 2:**
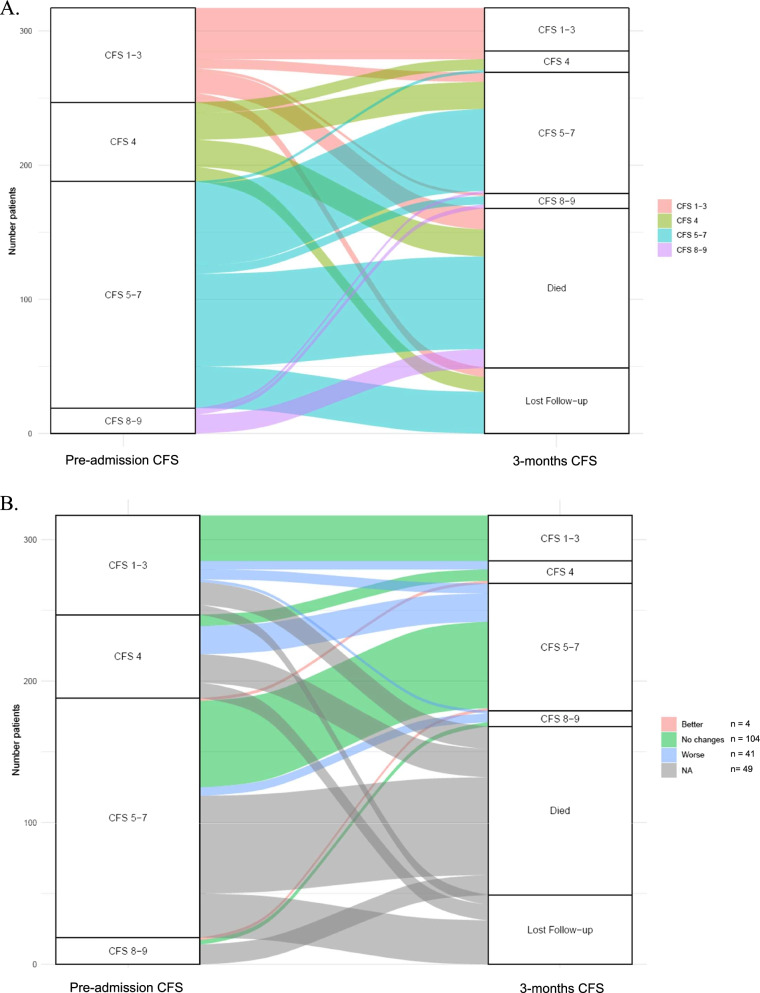
Transitions between frailty states 3 months after hospitalization in older COVID-19 survivors. **A**. Changes in frailty states from baseline (i.e., in the 2 weeks before acute admission) to 3-months follow-up among survivors of COVID-19 admission are illustrated. **B.** Patients who transitioned to better, worse frailty states and patients who had no transition in frailty state are shown. CFS = Clinical Frailty Scale

As shown in Fig. 2, transitions to worse frailty states occurred in 41 patients (26.8%) between pre-admission and 3 months. Few patients transitioned to better frailty states (4, 2.6%). No change in frailty state occurred in 104 patients (67.9%) between enrollment and 3 months.

## Discussion

Our study showed that 3-months’ functional decline occurred in one-third of patients and that the factors independently associated with functional decline were length of hospital stay, COVID-19 complications, age, history of stroke and depressive semiology. Furthermore, one half of patients experienced long term COVID-19 symptoms and 27% worsened frailty status at 3 months.

### Functional decline

In our study, 36% of patients lost at least 1 point on Katz ADL, which is consistent with published studies. Indeed, functional decline after COVID-19 was estimated between 27 and 54% in older patients [13, 14]. This occurrence varies greatly among studies populations and according time to assessment. A loss of 1 point of ADL is clinically significant with a significant impact on the patient’s life and worsen the burden on the caregiver [30].

Our results highlight the importance of comorbidities as previously described [31, 32]. Even though comorbidity score was not independently associated with functional decline in our study, stroke and depression were positively significant which underline the importance of analyzing each comorbidity in detail and not only with a score.

Age was also associated with functional decline as it was described after an hospitalization for an acute medical problem [33, 34] and after hospitalization for COVID-19 infection: in a study published by Siyi Zhu et al. with more than 400 younger patients (median age 49 y.o) [35] and in multicenter cohorts with older adults [32, 36]. To our knowledge, our study was the first to describe the association between acute complications of COVID-19 and functional decline which can be explained by the cumulative effect of acute medical illness on functional capacity [33]. And this results can explain why several studies have found significant association between length of hospitalization for a COVID-19 infection and functional decline [31, 36] as in ours. To our knowledge, only one published study from Carrillo-Garcia et al. described factors associated with 3-months functional decline after an hospitalization for COVID-19 infection in frail older adults [13]. Independent factors associated with 3-months functional decline were hospital acquired functional decline, hypoalbuminemia < 30 g/L and need for re-hospitalization. Contrary to our study, they didn’t find a significant link with length of stay or any comorbidity or the severity of the disease. These differences can be explained by the use of a different disability score (Barthel index instead of Katz ADL), and by differences in the inclusion criteria (age > 70).

Patients with ADL-decline were more likely discharged in rehabilitation center. Geriatrician are aware that patients with new or additional ADL dependence at the time of hospitalization discharge have higher risk to fail to regain independence, and that the ability to recover in the months following discharge are related to their pre-admission status (comorbidities, frailty). Those patients were therefore more often discharged in a rehabilitation center to do the best to prevent it or at least limit its magnitude.

Many studies evaluate immediate functional decline at hospital discharge after COVID-19 [32, 35, 37]. We thought it would be more relevant to evaluate functional status at distance because hospitalization itself could have caused functional or cognitive decline in context of isolation due to sanitary restriction [38]. Moreover, our choice to evaluate functional status at 3 months after discharge rather than another delay seems relevant with regard to actual definition of post-COVID-19 syndrome or chronic COVID-19: “which includes symptoms and abnormalities persisting or present beyond 12 weeks of the onset of acute COVID-19 and not attributable to alternative diagnoses” [39].

### Long term symptoms

A systematic review and meta-analysis from Leon-Lopez et al. estimated that 80% of the infected patients with SARS-CoV-2 developed one or more long-term symptoms [18], which is nearly twice as much as in our study. All included studies had mean age around 60 years old and to our knowledge, there is no published study about long term symptoms in specific geriatric population. Also, it is possible that fewer symptoms were reported since it was a purely declarative collection: reporting and recall bias are recurrent, in connection with the cognitive disorders prevalent in our population. In addition, some symptoms are sometimes not felt by patients, for example: less dyspnea may be reported because of less physical activity. Consistent with actual literature, fatigue was the most frequent long-term symptom [18]. Compared to other studies, fewer patients report loss of taste and smell (5–8% versus 15–20%) [40]. Recently, growing media attention is being given to the concept of “COVID-19 brain fog”, a controversial and non-specific mental syndrome after COVID-19 infection which can include various neuropsychological symptoms such as fatigue, concentration difficulties, inattention, neurocognitive disorders, memory difficulties [41]. Forty percent of the patients of our study had new memory difficulties or progression of neurocognitive disorders 3 months after discharge, similar to other studies [40]. Consistent with the medical literature, 30% of our patients experienced delirium in the acute phase of COVID-19 infection [42]. Delirium is known to be an omen of long-term cognitive disorders, such as increased risk for future dementia or faster decline in patients with preexisting dementia [43–45].

### Transition in frailty states

Among those with frailty at follow-up, 30% were not frail before COVID-19 disease, and 27% experienced a transition to a worse frailty state. There is no other study analyzing frailty evolution after COVID-19. A large number of studies showed that frailty assessed by CFS is a prognostic tool for COVID-19 short- and mid-term mortality regardless of age and comorbidities [46–51]. The concept of frailty and its different approaches describe a dynamic and intermediate state, characterized by diminished capacity to respond to stressors due to a reduced functional reserve [52, 53]. Acute or chronic stress and diseases, low activity level, poor nutritional status are substantial stressors that may interfere with the physiological homeostasis and thus trigger or accelerate a frailty status [52–54]. Transition in frailty status was previously described after critical illness and acute diseases [24]. However, results cannot be compared due to different study setting influencing frailty results with a mean age around 60, and more organ failure in ICU studies. Besides, our study underlines the existence of a significant association between functional decline and newly acquired or worsened preexisting frailty.

### Limitations

Some limitations of this study should be noted. First, it is a single-center study which reflects local geriatric care practices as in hospital admissions during these COVID-19 waves. Secondly, our study may show a loss of statistical power due to the few included patients. As detailed in the methods part, the sample size calculation (SSC) was not possible when we started the study. The SSC varies between to 317 patients to include (considering a 80% target power, a 2-sided alpha of 0.05, a 30% COVID-19 mortality rate in acute geriatric ward and pejorative 10% follow-up withdrawal) as new data details post-COVID functional decline between 30 to 50% depending on the time of assessment and the settings of recent published studies [13, 14]. Third, there is an important missing data for long-terms symptoms and frailty transition. Indeed, these missing data were inherent in the design of the study and difficulties of follow-up with extremely frail patients. Due to the prevalence of cognitive disorders (50%), declarative collection of long term symptoms may lead to reporting and recall bias. For patients with cognitive decline, data were systematically collected with the caregiver phone interviews. Due to the constant evolution of knowledge as the pandemic progresses, our initial standardized geriatric assessment framework did not include specific questions on long term symptoms, which is partly responsible for the missing data during the follow-up. However, the assessment of the primary endpoint was possible for 190 patients out of the 198 survivors which represents only 4% of lost to follow-up.

Finally, our results showed that, unexpectedly, approximately 64% of our patients have no functional decline 3 months after their hospitalization for COVID-19. The COVID-19 disease, by its acute severity in this population (28% of deaths during hospitalization and 35% at 30 days) may select the least vulnerable patients, leading to a potential survivorship bias. Characteristic of dead patients are detailed in Table 2S in supplemental data in order to present the analyzed population. Another explanation would be the presence of a scale attenuation effect: this population is composed of frail older patients with low pre-admission ADL scores leading to more difficulties to demonstrate a difference. This floor effects not specific to the Katz scale and can also be found with other functional status scales [55, 56]. Finally, it can be assumed that some patients were lost to follow-up secondary to altered health status that did not allow them to go to a geriatric consultation.

This study has many strengths. In particular, the availability of disability data prior to the onset of COVID-19 (a reflection of the patient’s usual functional and instrumental status) is one of the strong points of the study. Indeed, in usual practice, geriatricians are used to assess pre-admission frailty and pre-admission functional status in order to establish a personalized hospital care plan.

## Conclusion

In our study, the COVID-19 infection was associated with a functional decline in at least one third of the survivors. A half of patients had long term symptoms and 27% worsened frailty status. Factors independently associated with functional decline were history of stroke, depressive semiology, COVID-19 complications, age and length of hospital stay. Thus, these results encourage practitioners to establish discharge personalized care plan based on a multidimensional evaluation including geriatric and frailty assessment and in parallel based on severity assessment. Furthermore, specific geriatric follow-up is needed for these patients in order to propose long term personalized care plan. It would be interesting to carry out this study in a population of older adults with a COVID-19 infection occurring after a complete vaccination schedule against SARS-Cov-2. If we know that vaccination allows a clear reduction in mortality, the transition in frailty status and loss of functional status in these COVID-19 patients are unknown.

## Supplementary Information

**Additional file 1: Table 1S.** Univariable analysis of factors associated with functional decline. **Table 2S.** Overall COVID-19 survivors and dead patients’ characteristics.

## Data Availability

The datasets used and/or analyzed during the current study are available from the corresponding author on reasonable request.

## Abbreviations

AD: Katz Activities of Daily Living
BMI: Body Mass Index
COVID-19: Coronavirus Disease 2019
CCI: Charlson Comorbidity Index
CFS: Rockwood Clinical Frailty Scale
CI: Confidence Interval
FiO2: Fraction of inspired oxygen
IADL: Instrumental Activity of Daily Living of Lawton
ICU: Intensive Care Unit
OR: Odds Ratio
PCR: Polymerase Chain Reaction
SARS-CoV-2: Severe Acute Respiratory Syndrome Coronavirus 2
SD: Standard Deviation
SOFA: Sequential Organ Failure Assessment Score
