# Supplementary material for: Functional decline, long term symptoms and course of frailty at 3-months follow-up in COVID-19 older survivors, a prospective observational cohort study
Source: BMC Geriatr. 2022 Jun 30;22:542. doi: 10.1186/s12877-022-03197-y (PMC9244035; doi:10.1186/s12877-022-03197-y)
Supplement: Supplementary file 1 — Additional file 1: Table 1S. Univariable analysis of factors associated with functional decline. Table 2S. Overall COVID-19 survivors and dead patients’ characteristics. [file 12877_2022_3197_MOESM1_ESM.docx]

Baseline data collection

Clinical features were collected at admission with detailed symptoms and vital signs. Clinical and biological factors were collected in the clinical history: CRP, hepatic or renal abnormalities at admission, serum albumin levels at admission (normal>35 g/L, mild hypoalbuminemia 30–35 g/L and severe hypoalbuminemia<30 g/L) and other biological markers (troponin, ferritin, lymphopenia, anemia, thrombocytopenia); COVID-19 compatible abnormalities on CT-scan and their extension on the lung parenchyma.

Clinical severity parameters: Sepsis related Organ Failure Assessment (SOFA) score, Ordinal Scale for Clinical Improvement (OSCI), the oxygen support with maximal flow used and total duration of support, the occurrence of medical complications (such as pulmonary embolism, secondary bacterial infection, acute respiratory distress syndrome (ARDS), acute pulmonary edema, acute renal failure).

Drugs introduced in the acute phase were collected: type of anticoagulation therapy, use of corticotherapy.

At discharge, length of hospital stay and patient transfer to rehabilitation center and institutionalization were collected.

***Table 1S:*** Univariable analysis of factors associated with functional decline

|  |  | **OR [95%CI]** | **P value** |
| --- | --- | --- | --- |
| **Demographic** | Male | 0.76 [0.40; 1.42] | 0.390 |
|  | Age | 1.08 [1.02; 1.15] | 0.005 |
|  | Nursing home | 1.32 [0.72; 2.41] | 0.380 |
|  | BMI | 0.92 [0.86; 0.99] | 0.028 |
| **Comorbidity** | CCI | 1.13 [0.97; 1.32] | 0.130 |
|  | Chronic respiratory insufficiency | 0.74 [0.16; 2.76] | 0.670 |
|  | Cancer or hemopathy | 0.96 [0.5; 1.80] | 0.890 |
|  | Stroke | 4.54 [2.12; 10.2] | < 0.001 |
|  | Diabetes | 1.46 [0.66; 3.14] | 0.340 |
|  | Coronaropathy | 0.98 [0.49; 1.91] | 0.960 |
|  | High blood pressure | 1.97 [0.98; 4.15] | 0.056 |
|  | History of cardiac surgery | 0.37 [0.06; 1.49] | 0.180 |
|  | Chronic kidney disease stage 4 or 5 | 0.65 [0.20; 1.81] | 0.420 |
| **Geriatric assessment** | ADL | 0.97 [0.81; 1.16] | 0.720 |
|  | IADL | 0.64 [0.50; 0.81] | < 0.001 |
|  | CFS | 1.23 [1.02; 1.50] | 0.028 |
|  | Cognitive disorder | 2.12 [1.15; 3.95] | 0.015 |
|  | Prior fall | 2.52 [1.29; 5.07] | 0.006 |
|  | Polymedication | 1.13 [1.03; 1.23] | 0.006 |
|  | Depressive semiology | 2.02 [0.97; 4.23] | 0.062 |
| **Treatment before admission to hospital** | Angiotensin-converting enzyme (ACE) inhibitor. | 0.71 [0.36; 1.38] | 0.320 |
|  | Insulin | 1.55 [0.48; 4.87] | 0.450 |
|  | Anticoagulant therapy | 1.06 [0.54; 2.04] | 0.870 |
| **Clinical features** | Respiratory signs | 1.44 [0.70; 3.06] | 0.330 |
|  | Fever | 1.44 [0.79; 2.64] | 0.230 |
|  | Diarrhea | 1.89 [0.96; 3.73] | 0.065 |
|  | Dehydration | 2.07 [1.10; 3.92] | 0.024 |
| **Biological parameters** | CRP | 1.00 [1.00; 1.01] | 0.680 |
|  | Albumin | 0.96 [0.90; 1.03] | 0.240 |
|  | Creatinin | 1.00 [0.99; 1.00] | 0.920 |
|  | Troponin | 1.01 [1.00; 1.02] | 0.130 |
|  | D-dimer | 1.00 [1.00; 1.00] | 0.920 |
|  | Lymphocytes | 1.04 [0.90; 1.21] | 0.550 |
|  | Platelets | 1.00 [1.00; 1.01] | 0.028 |
|  | Neutrophils | 1.05 [0.93; 1.18] | 0.440 |
|  | Vitamin D | 1.00 [0.97; 1.02] | 0.780 |
| **Severity** | Length of stay (day) | 1.06 [1.02; 1.09] | 0.001 |
|  | Length of oxygen supply (day) | 1.01 [0.97; 1.05] | 0.540 |
|  | Maximal oxygen supply (L/min) | 1.02 [0.95; 1.10] | 0.520 |
|  | SOFA score (hospital admission) | 1.08 [0.86; 1.36] | 0.490 |
|  | OSCI score (hospital admission) | 1.12 [0.69; 1.86] | 0.640 |
| **In-hospital complications** | Complications* | 2.48 [1.24 ; 5.22] | 0.012 |
|  | Paroxysmal FA | 0.87 [0.12; 4.68] | 0.880 |
|  | Pulmonary oedema | 1.29 [0.48; 3.38] | 0.600 |
|  | Delirium | 1.52 [0.78; 2.97] | 0.220 |
|  | Acute kidney failure | 1.62 [0.68; 3.85] | 0.270 |
|  | Secondary infection | 1.85 [1.01; 3.42] | 0.047 |
|  | Acute urine retention | 1.18 [0.15; 7.41] | 0.860 |
|  | ARDS | 0.35 [0.02; 2.24] | 0.290 |
|  | Thrombosis | 0.32 [0.05; 1.27] | 0.110 |
| **In-hospital treatment** | Corticosteroid drugs | 1.13 [0.62; 2.06] | 0.690 |
|  | Oxygen supply | 1.28 [0.70; 2.38] | 0.420 |
|  | Oxygen flow >6L/min | 0.53[0.66; 2.95] | 0.370 |
|  | Antibiotic therapy | 2.06 [1.09; 4.01] | 0.025 |
|  | Curative anticoagulation | 0.93 [0.50; 1.70] | 0.810 |

Complications*: Thrombosis or acute kidney failure with dehydration or diarrhea or secondary infection

Table 2S: Overall COVID-19 survivors and dead patients’ characteristics

|  |  | **COVID patients**  **N= 318** | **Survivors**  **N= 198** | **Dead**  **N= 120** | **P value** |
| --- | --- | --- | --- | --- | --- |
| **Demographic and anthropometric** | Male | 136 (43%) | 71 (36%) | 65 (54%) | 0.001 |
|  | Age (years) | 87 (83.0-91.0) | 86 (82.0-90.0) | 88 (83.0-93.0) | 0.024 |
|  | Nursing home | 133 (42%) | 76 (38%) | 57 (48%) | 0.110 |
|  | BMI (kg/m^2^) | 24.6 (21.1-27.9) | 24.6 (21.9-28.2) | 24.8 (20.0-27.0) | 0.200 |
| **Comorbidities** | CCI | 2 (1.0-4.0) | 2 (1.0-3.0) | 3 (2.0-4.0) | <0.001 |
|  | Chronic respiratory insufficiency | 23 (7.3%) | 11 (5.6%) | 12 (10%) | 0.140 |
|  | Stroke | 68 (21%) | 37 (19%) | 31 (26%) | 0.130 |
|  | Diabetes | 57 (18%) | 32 (16%) | 25 (21%) | 0.300 |
|  | Coronaropathy | 86 (27%) | 51 (26%) | 35 (29%) | 0.500 |
|  | Hypertension | 240 (75%) | 145 (73%) | 95 (79%) | 0.200 |
|  | Chronic kidney disease stage 4 or 5 | 38 (12%) | 18 (9.1%) | 20 (17%) | 0.044 |
| **Geriatric assessment** | Pre admission ADL | 4.5 (3.0-6.0) | 5 (3.0- 6.0) | 4.5 (2.5- 5.5) | 0.031 |
|  | Pre admission IADL | 1 (0-3.0) | 1 (0- 3.0) | 1 (0- 2.0) | 0.085 |
|  | Pre admission CFS | 5 (4-6) | 5 (3- 6) | 6 (4- 7) | <0.001 |
|  | Cognitive disorder | 151 (51%) | 98 (51%) | 53 (51%) | >0.900 |
|  | Prior fall | 147 (59%) | 99 (58%) | 48 (62%) | 0.500 |
|  | Polymedication | 8 (5.0-10.0) | 7 (5.0- 10.0) | 8 (6.0- 11.0) | 0.010 |
|  | Depressive semiology | 52 (16%) | 38 (19%) | 14 (12%) | 0.079 |
| **Clinical features** | Respiratory signs | 264 (83%) | 150 (76%) | 114 (95%) | <0.001 |
|  | Fever | 167 (53%) | 106 (54%) | 61 (51%) | 0.700 |
|  | Delirium | 95 (30%) | 48 (24%) | 47 (39%) | 0.005 |
|  | Dehydration | 115 (36%) | 60 (30%) | 55 (46%) | 0.004 |
|  | Diarrhea | 68 (21%) | 49 (25%) | 19 (16%) | 0.060 |
| **Severity** | Length of stay (day) | 10 (6- 16) | 11 (7- 17) | 9 (5- 15) | 0.079 |
|  | Length of oxygen supply (day) | 4 (0- 9) | 2 (0- 8) | 6 (3- 10) | <0.001 |
|  | Maximal oxygen supply (L/min) | 4 (0- 12) | 2 (0- 4) | 15 (4.2- 15) | <0.001 |
|  | SOFA score (hospital admission) | 1 (0- 2) | 0 (0- 1) | 2 (1- 3) | <0.001 |
| **In-hospital complications** | Patient with 1 complication or more | 218 (80%) | 133 (67%) | 85 (93%) | <0.001 |
|  | Paroxysmal FA | 17 (8.6%) | 6 (3%) | 11 (14%) | 0.037 |
|  | Acute kidney failure | 69 (35%) | 29 (14.6%) | 40 (49%) | <0.001 |
|  | Secondary infection | 150 (50%) | 85 (42.9%) | 65 (59%) | 0.021 |
|  | ARDS | 29 (14%) | 6 (3%) | 23 (28%) | <0.001 |
|  | Thrombosis | 21 (7%) | 12 (6%) | 9 (8.3%) | 0.500 |
| **Hospital treatments** | Corticosteroid drugs | 160 (50%) | 80 (40%) | 80 (67%) | <0.001 |
|  | Oxygen supply | 226 (71%) | 116 (59%) | 110 (92%) | <0.001 |
|  | Antibiotic therapy | 230 (72%) | 124 (63%) | 106 (88%) | <0.001 |
|  | Transfer in ICU | 6 (2.2%) | 2 (1.3%) | 4 (3.6%) | 0.200 |
